# Design of a High Precision Ultrasonic Gas Flowmeter

**DOI:** 10.3390/s20174804

**Published:** 2020-08-26

**Authors:** Jianfeng Chen, Kai Zhang, Leiyang Wang, Mingyue Yang

**Affiliations:** College of Metrology & Measurement Engineering, China Jiliang University, Hangzhou 310018, China; p1802085205@cjlu.edu.cn (J.C.); p1702085249@cjlu.edu.cn (L.W.); s1902080445@cjlu.edu.cn (M.Y.)

**Keywords:** ultrasonic gas flowmeter, the principle of time-difference method, data filtering, low-power measurement

## Abstract

Aiming at the problems of substantial pressure loss, small range ratio and contact measurement in traditional gas flowmeters, this paper designs a new type of data-filtering ultrasonic gas flowmeter. The flowmeter is composed of hardware circuits such as STM32F407 (ARM Cortex 32-bit microcontroller) main control chip and high-precision timing chip TDC-GP22 (time to digital converter). The software uses a new data-filtering algorithm combining Kalman filtering algorithm and arithmetic average algorithm to improve the measurement accuracy of ultrasonic gas flowmeter. Through experimental comparison, we find that the filtering algorithm effectively reduces the measurement error of the system. Within the flow range of 0.025–4 m^3^/h, the maximum relative error of the system measurement is 2.7404%, which meets the national standard for the measurement error of the 1.5-level instruments. Moreover, it reduces the zero-drift to about one half of the original, which significantly improves the stability of the system. The gas flowmeter has the characteristics of high accuracy, good stability, low power consumption, and the overall performance is significantly improved.

## 1. Introduction

Nowadays, flow detection technology has been widely used in various fields of industrial production such as petroleum, chemical industry, energy, etc. [1,2]. As a flowmeter for detecting fluid, flowmeters can be divided into gas flowmeters and liquid flowmeters from the measurement medium. Traditional turbine flowmeters, vortex flowmeters, and orifice flowmeters play an important role in the measurement of liquid flow [3]. However, due to the particularity of the gas medium flow and the complexity of the problems encountered in the signal transmission in the gas, the severe issues in gas flow measurement still plague people [4]. In the field of gas measurement, most domestic gas meters currently used in the country are membrane gas meters. The metering principle of the traditional membrane gas meter is to introduce the gas into a metering chamber with a constant volume and discharge it when it is full. Through a specific transmission mechanism, the number of charges and exhaust cycles converts into a mass, which reflects in the counter of the gas meter on [5]. The measurement technology of membrane gas meter is mature, reliable measurement and stable quality. However, its interior is contact measurement. Due to long-term wear, the measurement accuracy, sensitivity and stability of the measuring element is reduced, and there are problems such as substantial pressure loss, small range ratio and difficult meter reading. It has been challenging to meet the needs of modern society and people’s daily life [6].

With the development of electronic information and the Internet of things technology, ultrasonic flowmeters have become a new type of intelligent instrument. Ultrasonic flowmeters achieve remote data transmission in combination with the Internet of things technology, solving the problem of field meter reading [7]. Compared with the traditional membrane gas meter, the ultrasonic gas flowmeter has the outstanding advantages of noncontact type, small pressure loss, wide range ratio, high accuracy, etc. [8].

In recent years, a large number of researchers have made significant achievements in the study of fluid pipeline structure and ultrasonic transducers [9,10]. In 2013, Zhao Xuesong conducted a flow field simulation on the pipeline of a mono ultrasonic flowmeter. The flow field characteristics of different shapes and sizes in the pipeline are studied, and the best tapered pipeline model is designed [11]. In 2015, Li Yuxi studied the influence of different installation methods of ultrasonic transducers on the measurement accuracy of flow meters and determined the optimal installation method of ultrasonic flow meters [12]. Moreover, the data filtering of the ultrasonic gas flowmeter mainly adopts the median filtering algorithm and wavelet algorithm to improve the measurement performance [13]. In 2018, Yao Ping used algorithms such as wavelet analysis and recursive average filtering to study how to improve the measurement accuracy of gas ultrasonic flowmeters in complex flow fields [14]. These research results have promoted the widespread application of ultrasonic gas flowmeters [15].

However, most of the existing ultrasonic gas flowmeters meet the measurement accuracy requirements in the high zone (From the transition flow Q_t_ (including Q_t_) to the maximum flow Q_max_: Q_t_ ≤ Q_i_ ≤ Q_max_) and the accuracy requirements of small flow in low zone (From minimum flow Q_min_ to transition flow Q_t_: Q_min_ ≤ Q_i_ < Q_t_) cannot be guaranteed [16]. To further improve the overall performance of the ultrasonic gas flowmeter, this paper designs a new data-filtering algorithm combining Kalman filtering and arithmetic average. The small flow in the low zone has especially high measurement accuracy, which significantly improves its measurement stability and can better meet the needs of practical applications.

## 2. Method of Time-Difference Measurement

### Principle of Time Difference Measurement

In this paper, the ultrasonic gas flowmeter adopts the principle of time-difference method. The time-difference method is to indirectly obtain the average flow velocity of the fluid medium by measuring the transmission time interval of the ultrasonic signal in the fluid medium in the forward and reverse directions [17].

**Figure 1:**
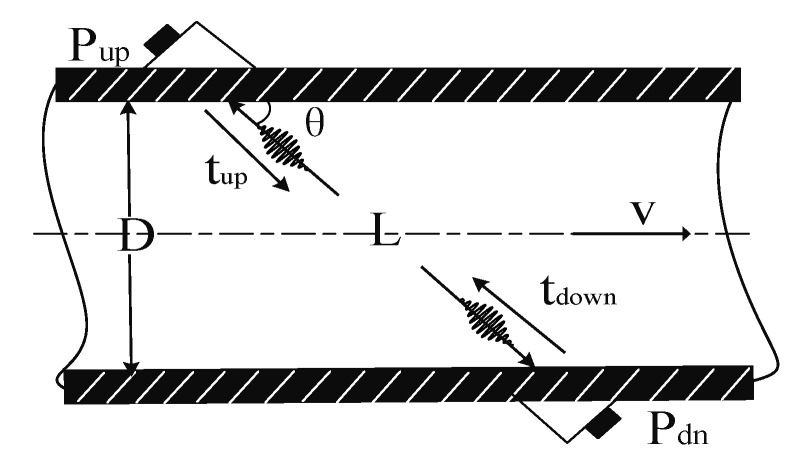
Schematic diagram of the principle of time-difference method.

Figure 1 shows the measurement principle of the time-difference method [18]. Transducer P_up_ is a forward-flow transducer, and P_dn_ is a reverse-flow transducer. The fluid flows from left to right in Figure 1 as a positive flow direction. The installation angle of the transducers and the pipe is *θ*, the pipe diameter is *D*. The linear distance between the two transducers is *L*. The transmission speed of ultrasonic waves in a gas medium is *c*. The forward velocity of the gas is *v*. The upstream time transmitted by the P_up_ transducer and received by the P_dn_ transducer is [19]:

(1) $$t_{up}=\frac{L}{c+v\cos\theta }$$

The downstream time transmitted by the P_dn_ transducer and received by the P_up_ transducer is:

(2) $$t_{down}=\frac{L}{c-v\cos\theta }$$

From Equations (1) and (2), through the downstream time transmitted $t_{down}\;$ minus the upstream time transmitted $\;t_{up}$, the forward and reverse flow time difference Δ*t* of the ultrasonic signal transmission can be obtained.

(3) $$\Delta t=t_{down}-t_{up}=\frac{2Lv\cos\theta }{c^{2}-v^{2}\cos^{2}\theta }$$

Because the sound velocity c of the ultrasonic wave is much greater than the flow velocity v of the gas, so $c^{2}$ much larger than $v^{2}\cos^{2}\theta$, the Equation (3) can be approximated as:

(4) $$v=\frac{\Delta tc^{2}}{2L\cos\theta }$$

The linear velocity *v* of the gas can be obtained by Equation (4). When measuring the instantaneous flow rate of gas, it is necessary to use the average surface velocity to calculate. According to the relevant knowledge of fluid mechanics, the average surface velocity has different correction coefficients according to different states of the fluid. Let the correction coefficient be *K*, then the instantaneous flow *Q* of the gas is:

(5) $$Q=K\times S\times v=\frac{K\pi D^{2}}{4}v$$

where *S* is the cross-sectional area of the pipeline; *K* is the instrument factor, which is related to the Reynolds number of the fluid state.

It can be seen from Equations (4) and (5), under the condition of ensuring the accuracy of other fixed quantities, accurate measurement of the time difference Δ*t* of the forward and reverse flow is the key to ensure the system’s metering accuracy [20].

## 3. Hardware Design and Signal-Processing Process

### 3.1. Overall Hardware Design

**Figure 2:**
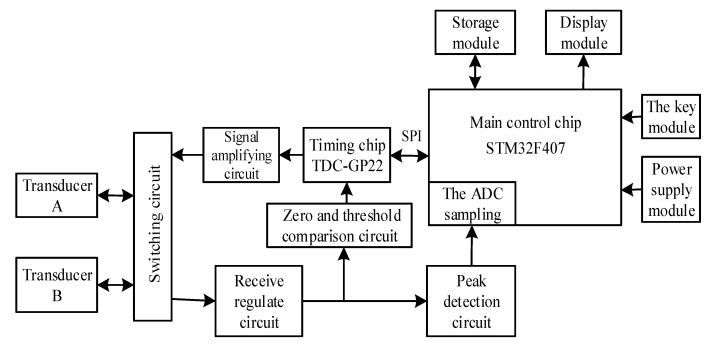
System hardware block diagram.

Figure 2 shows the system hardware block diagram of the ultrasonic gas flowmeter. The system is mainly composed of a control chip, a timing chip, an excitation signal amplifying circuit, a receiving conditioning circuit, an LCD (liquid crystal display) module and a power module.

This system uses a 32-bit single-chip STM32F407 as the calculation and control module, which meets the requirements of high operation accuracy and low-power-consumption measurement. This system selects the TDC-GP22 high-precision timing chip as the time measurement module. Through the SPI (serial peripheral interface) communication mode, the single-chip microcomputer controls the pulse generator inside the TDC-GP22 chip to generate an excitation signal and amplified by the amplifier circuit, which acts on the transmitting transducer to send out the transmit waveform and start the measuring time. Moreover, transmitting in the pipeline for some time, the transmit waveform is received by the receiving transducer. After being amplified and filtered, the ultrasonic echo signal is sent to the threshold comparison circuit, and the zero-crossing generates a signal to stop timing. Finally, it sends to the TDC-GP22 timing chip to complete the time measurement. The TDC-GP22 sends an interrupt signal to the microcontroller after the measurement is completed. The microcontroller reads the time-difference measurement result and brings into the flow calculation formula to display and store the flow data.

### 3.2. TDC-GP22 Circuit Generates an Excitation Signal

**Figure 3:**
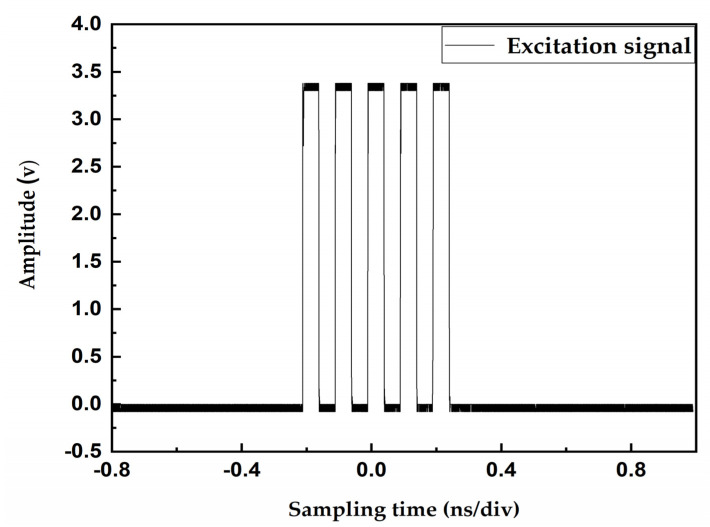
High-precision timing chip (TDC-GP22) circuit generates an excitation signal.

The transit time of the ultrasonic gas flowmeter is at the nanosecond level; the TDC-GP22 can measure at 90-picosecond resolution in the single-precision mode and at 45-picosecond resolution in double precision mode. All meet our requirements for time-difference measurement accuracy. To reduce power consumption, we choose a single-precision measurement mode. The TDC-GP22 high-precision timing chip used in this paper integrates a pulse-generating circuit, which greatly simplifies the configuration of peripheral circuits. The internal logic gate delay is used to measure the time interval to ensure the accuracy of the measurement. Figure 3 shows the excitation signal generated by the TDC-GP22 pulse-generating circuit.

### 3.3. The Amplified Excitation Signal by the Amplifier Circuit

**Figure 4:**
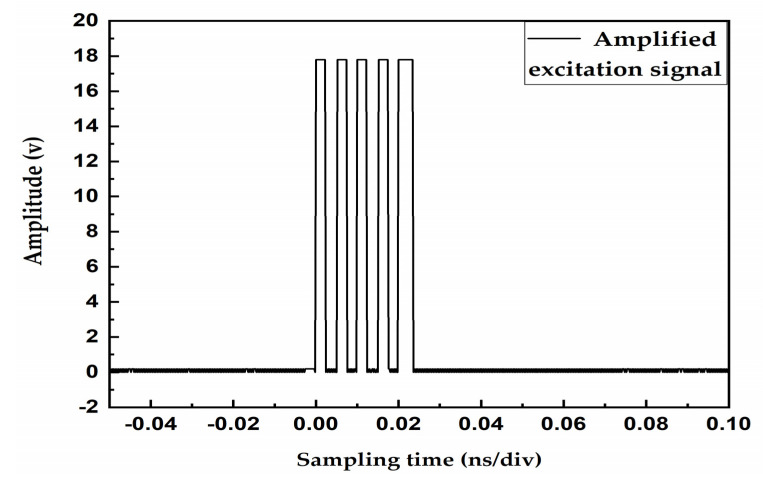
Amplified excitation signal by the amplifier circuit.

Figure 3 shows that the amplitude of the excitation signal is 3.3 V. In actual design—because the ultrasonic signal generated by low-amplitude excitation is attenuated severely in the gas medium—an excitation source with an amplitude of more than 15 V is generally required to excite the gas ultrasonic transducer. Therefore, we used the TPS61085 (Booster converter) boost chip to amplify the original signal. Figure 4 shows that the amplifier circuit boosts the 3.3 V voltage of the initial excitation signal to 18 V, which acts on the transmitting transducer to send out the transmit waveform.

### 3.4. The Receiving Circuit Processes the Echo Signal

**Figure 5:**
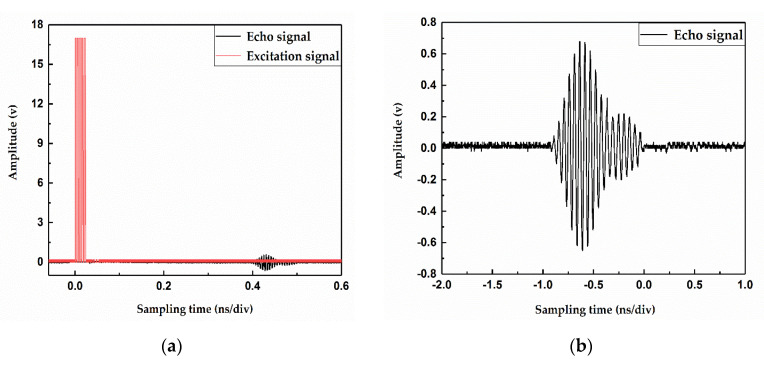
(**a**) Signal processed before the receiving circuit; (**b**) signal obtained by the receiving circuit.

After propagating through the fluid medium, the transmit waveform signal is received by the receiving transducer. Figure 5a shows the original echo signal generated by the receiving transducer has an amplitude of several tens of millivolts, and it contains some noise interference signals. To obtain a stable and pure echo signal, the received original signal needs to be conditioned [21]. Therefore, we use the low-noise and high-precision op amp OPA320 (precision operational amplifier) to amplify the weak echo signal containing noise and then pass the active band-pass filter circuit to filter the amplified high and low-frequency interference signals. Finally, a stable and pure echo signal is obtained. Figure 5b shows the echo signal obtained after amplification and filtering by the receiving circuit.

### 3.5. Threshold and Zero-Crossing Comparison Circuit Generates Stop Timing Signal

**Figure 6:**
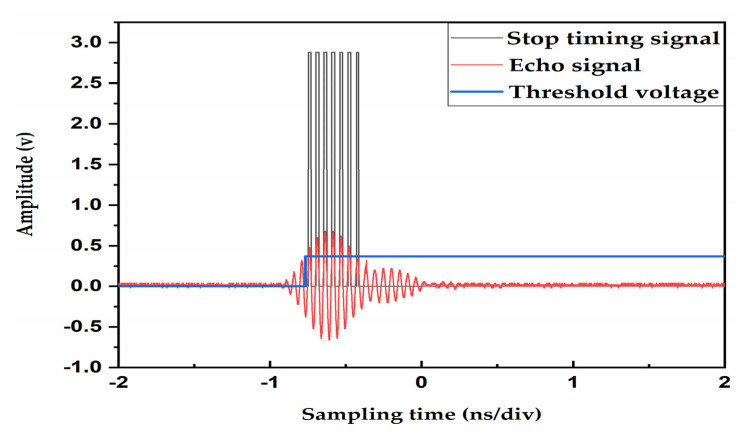
Threshold and zero-crossing comparison circuit generate the stop-timing signal.

To avoid some interference signals will also trigger the TDC-GP22 chip timing. We first use a threshold comparison circuit to set a threshold voltage. When the amplitude of the echo signal is higher than the threshold voltage, the enable pin of the zero-crossing comparison circuit is triggered and generates a stop-timing signal and sends it to the stop pin of TDC-GP22 to complete timing [22]. In this way, the interference before the useful signal is shielded by a combination of threshold and a zero-crossing comparison circuit. Figure 6 shows the stop-timing signal generated by the threshold and zero-crossing comparison circuit.

## 4. Software Design

### 4.1. Software System Design

**Figure 7:**
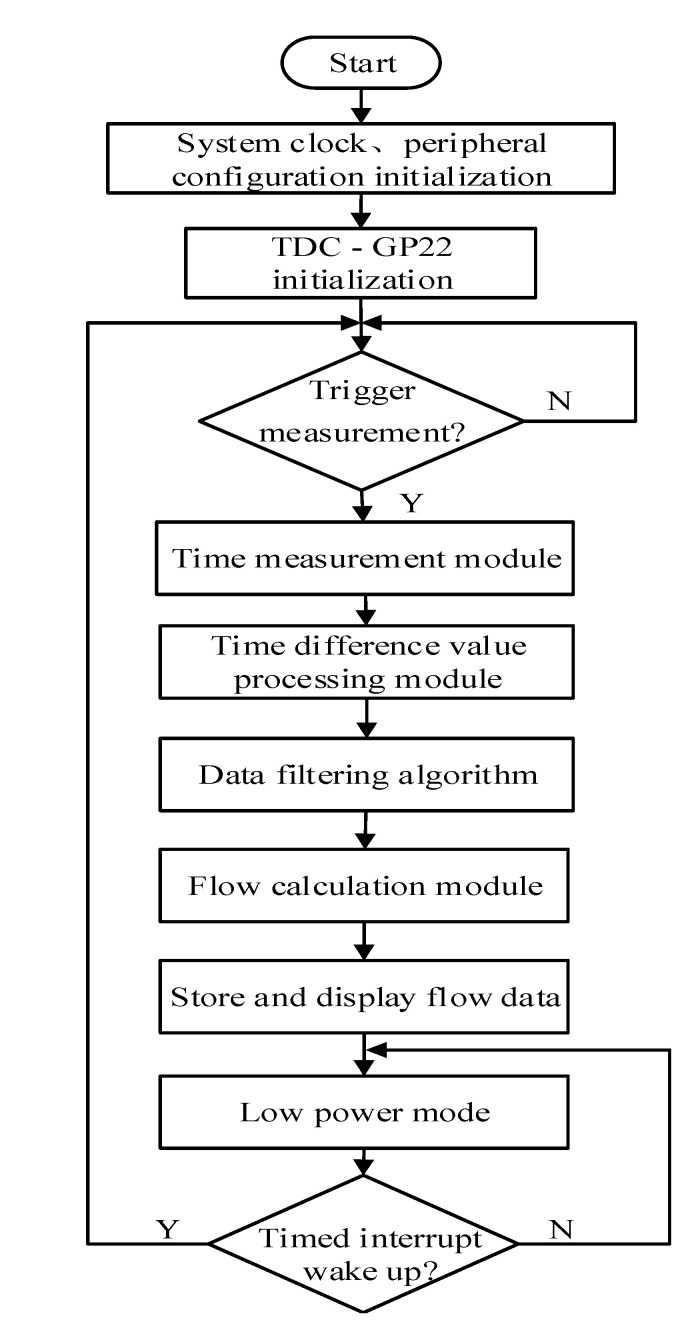
System software flow chart.

Figure 7 shows the flow chart of ultrasonic gas flowmeter measurement. When the measurement starts, the single-chip microcomputer needs to be initialized, including initializing the corresponding peripheral configuration and system clock and then configuring the registers of the TDC-GP22. The single-chip microcomputer sends an instruction to start measurement to the TDC-GP22 through the SPI communication method and simultaneously enters the time measurement program. After completing the upstream and downstream time measurement, it enters the time-difference processing module and then runs a filtering algorithm to process the time-measurement data. Finally, the single-chip microcomputer calculates the gas flow and send the data to the LCD. At the same time, the flow rate data are stored in the main control chip’s internal flash to prevent data loss after power failure. After completing measurement, the single-chip microcomputer enters the low power STANDBY mode and waits for the timer interrupt to wake up for the next measuring.

The ultrasonic gas flowmeter in this study is battery-powered, so its system must use low-power measurement. The power consumption of the STM32F407 microcontroller in STANDBY mode is less than 2.5 μA and the static power consumption of the TDC-GP22 timing chip is the only 2.2 μA. To reduce the measurement power consumption of the system, the sampling frequency of the system is related to the change of the gas flow rate. When the gas flow rate is considered to be stable, the sampling period sets to 1 s and the rest of the time is in a dormant state. When the gas flow rate changes, the system will increase the sampling frequency to reflect the amount of gas change.

### 4.2. Data Filtering

The purpose of data filtering is to eliminate random errors in the original data. A reasonable data-filtering algorithm must be based on a thorough study of the original data, taking into account data volatility, data distribution characteristics and sudden changes [23,24]. In the measurement of ultrasonic gas flowmeters, measurement deviations are caused by disturbing factors such as random noise and piping structure design errors. For the arithmetic average filtering algorithm, the smoother the filtering effect, the higher the lag of the algorithm and the poor ability to suppress random errors that occur in the system. For the application of the Kalman filter algorithm, it can achieve a strong ability to suppress the arbitrary interference appearing in the order and the filter effect will be worse when the response to sudden changes is realized. Therefore, in the case of frequent changes of gas flow rate, how to make the ultrasonic gas flowmeter respond quickly to the amount of gas change and achieve accurate measurement is another test of the filtering algorithm.

To balance the smoothing effect of filtering and the timeliness of data processing. In the design of the algorithm, it is necessary to discriminate whether the measurement deviations are caused by the interference of external random factors or the design error of the internal structure of the measuring pipe, then adopts different algorithms for different situations. Therefore, this study proposes a new data-filtering algorithm combining a Kalman filtering algorithm and an arithmetic averaging algorithm.

#### 4.2.1. Kalman Filtering Algorithm

Kalman filtering is a time–domain filtering method that is suitable for recursive solving. It can process the data obtained at each sampling instant immediately and based on the state estimates before that moment. The recursive equation gives the new state at any time estimate. Inflow measurement systems, the entire process can be represented by the following discrete models [25]:

(6) x(k)= Ax(k − 1) + Bu(k − 1) + w(k − 1)

(7) z(k) = Cx(k) + v(k)

where the *x*(*k*) is state variable and the gain matrix *A* is the state of *k* − 1 is linearly mapped to the state of *k* at the current time, matrix *B* represents the gain of the optional control input *u*(*k* − 1) and the random signal *w*(*k* − 1) is the process excitation noise, because the state of the process has no control inputs, *u* = 0, the matrix *C* represents the gain of the state variable *x*(*k*) against the measured variable *z*(*k*) and *v*(*k*) represents the observed noise [26].

The Kalman filtering algorithm is as follows:

1. Predict the current state:
  (8) $$\hat{x}\left(k\;|\;k\;-\;1\right)\;=\;A\left(k\;-\;1\right)\cdot \;\hat{x}\left(k-|\;k\;-\;1\right)$$
2. The covariance of prior estimation errors:
  (9) $$P\left(k|k\;-\;1\right)\;=\;A\left(k\;-\;1\right)P\left(k\;-\;1|k\;-\;1\right)A^{T}\left(k\;-\;1\right)\;+\;Q\left(k\;-\;1\right)$$
3. Gain calculation:
  (10) $$K\left(k\right)\;=\;P\left(k|k\;-\;1\right)C^{T}\left(k\right)\left[C^{T}\left(k\right)P\left(k|k\;-\;1\right)C\left(k\right)\;+\;R\left(k\right)\right]$$
4. Status estimate update:
  (11) $$\hat{x}\left(k|k\right)\;=\;\hat{x}\left(k|k\;-\;1\right)\;+\;K\left(k\right)\left[z\left(k\right)\;-\;C\left(k\right)\hat{x}\left(k|k\;-\;1\right)\right]$$
5. The covariance of posterior estimation error:
  (12) P(k|k) = [1 − K(k)C(k)]P(k|k − 1)

where $\hat{x}\left(k|k\;-\;1\right)\;$ is the prior estimate, $\hat{x}\left(k|k\right)\;$ is the posterior estimate and P(k|k − 1) is the covariance of the previous estimate error, P(k|k) is the covariance of the following estimation error, *K*(*k*) is the filter gain, *R*(*k*) is the observation noise covariance matrix, *Q*(*k* − 1) is the process excitation noise covariance matrix [27].

#### 4.2.2. Filter Algorithm Combining Kalman and Arithmetic Average

**Figure 8:**
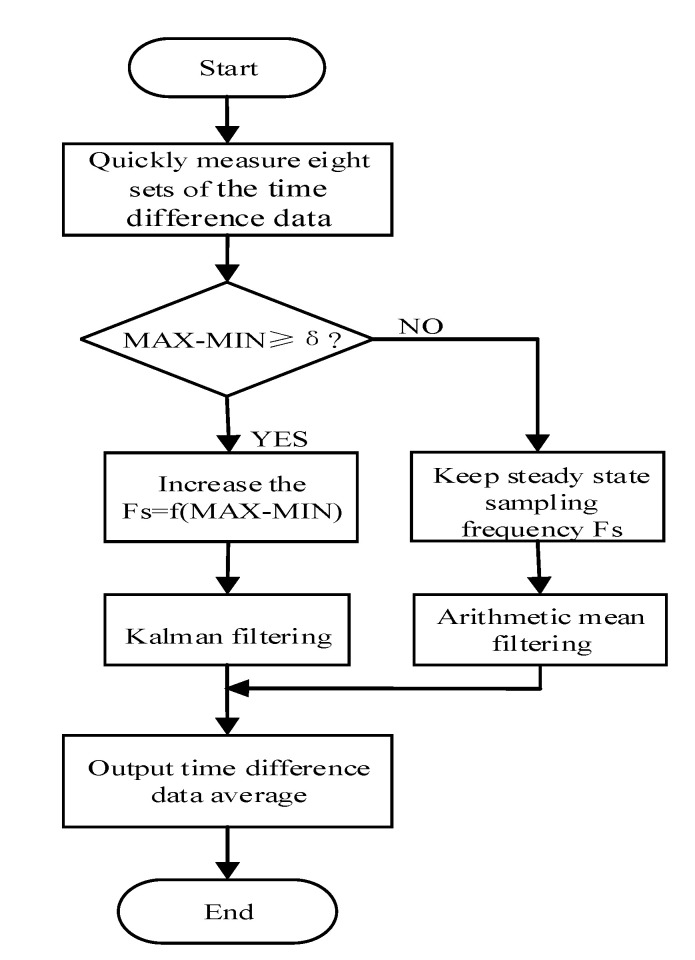
Data-filtering algorithm.

Figure 8 shows a data-filtering algorithm combining the Kalman filtering algorithm and the arithmetic averaging algorithm. First, the system quickly measures eight sets of time-difference data and determine the difference value between the maximum and minimum values. When the ultrasonic gas flowmeter detects that the difference value higher than or equal to a particular threshold δ, the data have a wide range at this time, the gas flow rate is considered to be in a rapidly changing stage due to external interference. Hence, the system increases the sampling frequency Fs to track the change of the gas flow rate. At this time, the system selects a Kalman filter algorithm to process the time-difference data. When the difference value is less than a particular threshold range δ, the range of data changes is small. The gas flow rate is considered to be relatively stable at this time. The sampling frequency Fs is reduced, and we select the arithmetic averaging algorithm to solve the problem that the internal structure design error of the actual pipeline affects the gas flow rate.

The data-filtering algorithm can effectively improve the measurement accuracy of the system. It can also significantly reduce the zero-drift of the system. Especially in the measurement of small flow, the stability and measurement accuracy of the system was improved.

## 5. Results and Discussion

### 5.1. Experimental Device

**Figure 9:**
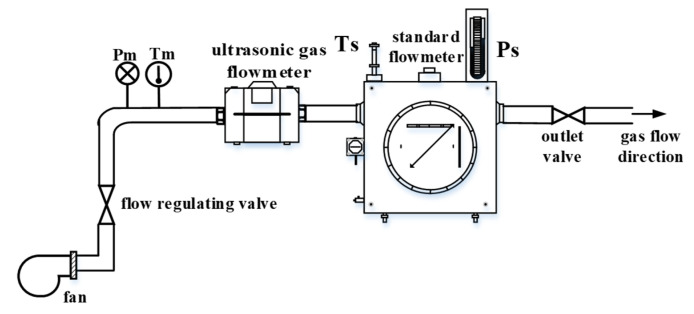
Schematic diagram of the experimental measurement system.

To test the effect of the comparison data-filtering algorithm, we use a gas flow measurement system for experiments. The schematic diagram of the experimental measurement system is shown in Figure 9, which is mainly composed of the standard flowmeter, the ultrasonic gas flowmeter, the pressure measurement sensor, the temperature measurement sensor, a flow regulating valve, a fan and an outlet valve.

In the experimental measurement, the ultrasonic gas flowmeter was installed on the pipe section of the verified flowmeter and the fan started to blow in air, and the air outlet valve was closed. After the pressure between the standard flowmeter and the verified flowmeter was consistent and the measurement system was stable. We opened the air outlet valve [28]. In the experiment, we adjusted the flow rate by adjusting the opening of the flow regulating valve. After running for some time, we read the flow data of the standard flowmeter and the verified flowmeter.

To verify the measurement performance of ultrasonic gas flow, it is necessary to test the instantaneous flow at different flow points. The measuring range designed is 0.025–4 m^3^/h in this study. According to “JJG-1030-2007 Verification regulation of ultrasonic flowmeters”, the detection flow points are Q_min_, Q_t_, 0.4 Q_max_, Q_max_, for each flow point test is no less than 3 times; the experimental results are shown in Table 1.

### 5.2. Zero-Drift Discussion

Zero-drift means that the flow rate is zero under the test conditions: the pipe section is filled with air, and the inlet and outlet of the pipe are sealed. Therefore, the time-difference data of the zero-drift is continuously collected when the flow rate is zero.

Theoretically, when the flow rate of the fluid is zero, the transmission time of the ultrasonic signal in the forward and reverse directions is equal. Actually, it is disturbed by factors such as pipe structure, the performance difference of upstream and downstream transducers, which causes the time difference to not be zero, so the calculated flow rate is not zero, and it will cause errors in the actual flow measurement. Especially for the measurement of small flow, because the time difference of the small flow is close to the time difference of the zero-drift, the error of the measured flow result is greater. Therefore, the zero-drift phenomenon will affect stability and accuracy of the ultrasonic flowmeter measurement.

**Figure 10:**
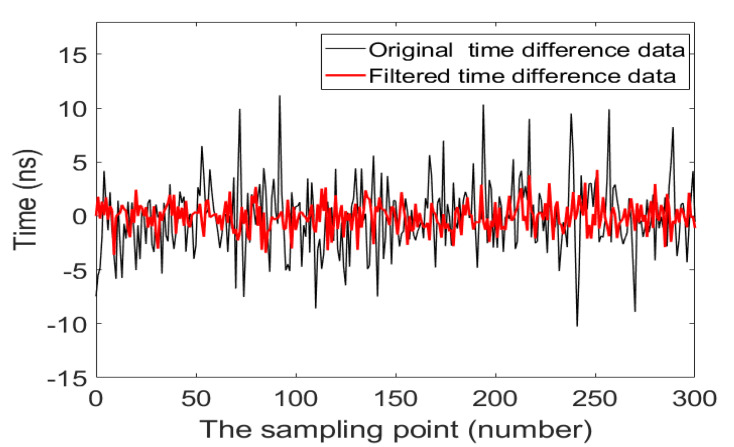
Comparison of zero-drift before and after data filtering.

To compare the effect before and after the data-filtering algorithm, we sampled the original zero-drift time-difference data and the zero-drift time-difference data after data-filtering algorithm. We labeled them as Sample 1, Sample 2, respectively. As shown in Figure 10, the black line represents the original zero-drift data. It can be seen from figure that the zero-drift range of the system remains within ±10 ns. The thick red line represents the zero-drift data obtained after data filtering. The zero-drift data range of the system is within ±5 ns. By comparison, it can be seen that the scope of the zero-drift data becomes significantly smaller.

To describe the degree of dispersion of the zero-drift data after the data filtering, the definition of sample standard deviation is introduced:

(13) $$S\;=\;\sqrt{\frac{1}{n\;-\;1}\overset{n}{\underset{i\;=\;1}{\sum }}{\left(X_{i}\;-\;\bar{X}\right)}^{2}}$$

where *S* is the samples standard deviation, $X_{i}$ is the time-difference value of the zero-drift, $\bar{X}$ is the average value of samples and *n* is the number of samples.

Table 2 analyses the difference value between the maximum and minimum value and standard deviation of the two groups of samples. The difference value indicates the range of the sample data [29]. The smaller the scale of the sample data, the more stable the data; and the smaller the standard deviation, the lower the degree of sample dispersion [30]. It can be seen from Table 1 that the difference value and standard deviation of Sample 2 are the smallest, and the difference value of Sample 1 is the largest. The results in Table 1 show that the data of Sample 2 are the most stable and have the least dispersion.

From the above analysis, it can be concluded that the data-filtering algorithm can effectively reduce the zero-drift of the system. The smaller the change range of the zero-point drift data, the more stable the measurement result of the ultrasonic flowmeter, and the less affected by the interference of the actual measurement environment.

### 5.3. Experimental Data

**Figure 11:**
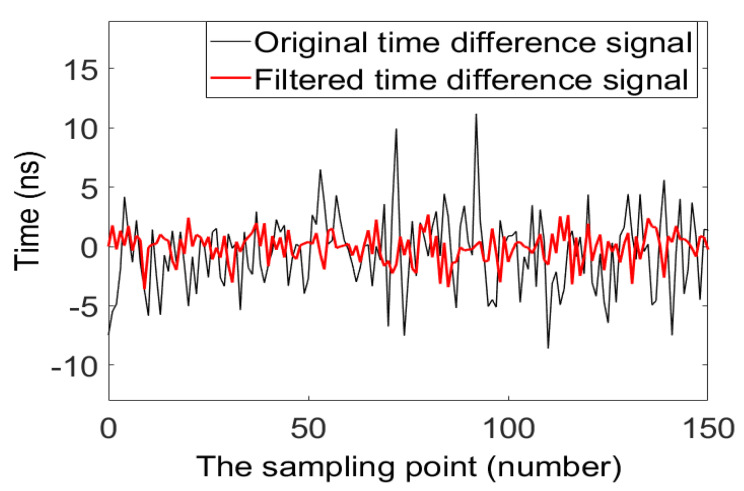
Comparison of time-difference signals before and after data filtering.

The range of the ultrasonic gas flowmeter is 0.025–4 m^3^/h, the transition flow Q_t_ is 0.4 m^3^/h, the range ratio R-value is 160, the accuracy level is 1.5 and the measuring pipe diameter is nominally diameter 20-mm in diameter. To verify and compare the effectiveness of data-filtering algorithms, we select two flow points distributed in the low zone (0.025 m^3^/h ≤ Q_i_ < 0.4 m^3^/h) and the high zone (0.4 m^3^/h ≤ Q_i_ ≤ 4 m^3^/h) and test multiple groups time-difference data. Three sets of data were selected for each flow point, as shown in Table 3 and Table 4, respectively. The time-difference data of a particular flow point is chosen randomly and the effect of the data-filtering algorithm is tested. The comparison of the results before and after the data filtering is shown in Figure 11.

This study uses the time-difference method to measure the flow rate of an ultrasonic gas flow meter. Combining Equations (4) and (5), it can be seen that the ultrasonic velocity *c* in a static fluid is taken as a constant. The installation angle *θ* of the transducer and the pipe and the linear distance *L* between the two transducers are directly measurable physical quantities, so the accurate measurement of the time difference between the forward and reverse flow can improve the measurement accuracy of the system. To verify the effect of the filtering algorithm and eliminate the influence of other physical constants on the verification results, this study compares the relative error data of the three sets of time difference before and after filtering and improves the measurement accuracy of the system by accurately measuring the time difference between the forward and reverse flow.

Figure 11 is a comparison of the effect of time-difference signals before and after data-filtering algorithm at a particular flow point. The black line represents the signal before data filtering, and the thick red line represents the signal after data filtering. By comparison, we can find that the time-difference signal after data filtering is smoother than the time-difference signal before data filtering. It shows that the filtered signal is less affected by the random interference.

### 5.4. Data Discussion

Table 2 and Table 3 show three sets of time-difference data before and after data filtering. The formula for calculating relative errors in table is:

(14) $$E\;=\;\frac{\Delta T}{T}\;\times \;100\%$$

where *E* is the actual relative error, generally given as a percentage, Δ*T* is the absolute error, that is the difference value between the measured value and the real value, and *T* is the real value. In the actual calculation, the average value is used instead of the real value.

It can be seen from Table 3 that when the flow point is in the high zone, the relative error of time-difference data is small; when the flow point is in the low zone, the relative error of time-difference data is large, and it is difficult to achieve accurate measurement. It can be seen from Table 4 that when the flow point is in the high zone, the relative error is small. Especially the relative error of the low zone flow is significantly reduced, which proves that the relative error of time-difference data after data filtering is smaller, and the measurement accuracy is higher.

To better compare the time-difference data in Table 3 and Table 4, the sample averages of the time-difference data of the low zone and high zone flow and the difference Δ*E* between the maximum and minimum of the relative error were calculated. The results are shown in Table 5.

By comparing the data in Table 5, it can be found that the relative error Δ*E* of a particular flow point in the low zone has decreased from 17.7258% before data filtering to 1.7101% after data filtering. The relative error Δ*E* of a particular flow point in the high zone decreased from 1.5303% before data filtering to 0.4260% after data filtering. The comparison shows that the data using the filtering algorithm is more stable and more accurate.

## 6. Conclusions

This paper designs a high-precision ultrasonic gas flow meter and analyses the signal-processing process of the ultrasonic gas flow meter. A data-filtering algorithm combining Kalman filtering and arithmetic averaging is proposed to improve the measurement accuracy and stability of the system.

The zero-drift of the ultrasonic flowmeter is an essential manifestation of the measurement performance of the entire system. The smaller the zero-drift is, the higher the measurement stability of the system is. Therefore, by comparing the effects of the data-filtering algorithm, it is evident that the zero-drift scope of the system is reduced from within ±10 ns to within ±5 ns. The zero-drift is about 1/2 of the original zero-drift of the system, which effectively improves the stability of the system measurement.

To verify the effectiveness of the data-filtering algorithm, it can be seen from the experimental comparison results that the relative error of the time-difference data after the data-filtering algorithm is reduced, especially the relative error of the small flow is reduced more obviously. Within the flow range of 0.025–4 m^3^/h, the maximum relative error of the system measurement is 2.7404%, which meets the national standard for the measurement error of the 1.5-level instruments. It shows that the measurement accuracy of the system is higher after data filtering, and its comprehensive measurement performance was significantly improved.

## Figures and Tables

**Table 1:** Flow experiment data at different flow points.

Average Value of the Standard Flowmeter (m^3^/h)	The Value of the Verified Flowmeter (m^3^/h)	Average Value of the Verified Flowmeter (m^3^/h)	Relative Error (%)
0.0250	0.0253	0.0257	2.7404
	0.0259		
	0.0258		
0.4000	0.3975	0.3933	−1.6731
	0.3922		
	0.3902		
1.6000	1.6477	1.6174	1.0901
	1.6233		
	1.5813		
4.0000	3.9530	3.9705	−0.7364
	3.9348		
	4.0238		

**Table 2:** Zero-drift data analysis.

Number	Difference between Maximum and Minimum (ns)	Standard Deviation (ns)
Sample 1	17.258	2.8873
Sample 2	7.893	1.3232

**Table 3:** Time-difference signal samples without data filtering.

Particular Flow Point in the Low Zone Time Difference Data (μs)	Relative Error (%)	Particular Flow Point in the High Zone Time Difference Data (μs)	Relative Error (%)
0.2978	−9.4558	1.6293	0.1332
0.3561	8.2700	1.6136	−0.8317
0.3328	1.1858	1.6385	0.6986

**Table 4:** Time-difference signal samples with data filtering.

Particular Flow Point in the Low Zone Time Difference Data (μs)	Relative Error (%)	Particular Flow Point in the High Zone Time Difference Data (μs)	Relative Error (%)
0.2625	−0.2407	1.5698	−0.1950
0.2612	−0.7347	1.5723	−0.0360
0.2657	0.9754	1.5765	0.2310

**Table 5:** Performance comparison with and without data filtering.

	Before Data Filtering	After Data Filtering
**Sample average of a particular flow point in the low zone (μs)**	0.3289	0.2631
**Sample average of a particular flow point in the high zone (μs)**	1.6271	1.5729
**Relative error Δ*E*** **in the low zone**	17.7258%	1.7101%
**Relative error Δ*E*** **in the high zone**	1.5303%	0.4260%
